# The Chemical Structure Properties and Promoting Biofilm Activity of Exopolysaccharide Produced by *Shigella flexneri*

**DOI:** 10.3389/fmicb.2021.807397

**Published:** 2022-02-04

**Authors:** Yinglong Song, Fenglian Ma, Mengying Sun, Guangqing Mu, Yanfeng Tuo

**Affiliations:** ^1^School of Food Science and Technology, Dalian Polytechnic University, Dalian, China; ^2^Dalian Probiotics Function Research Key Laboratory, Dalian Polytechnic University, Dalian, China

**Keywords:** *Shigella flexneri*, polysaccharide, structure identification, biofilm formation, matrix interaction

## Abstract

*Shigella flexneri* is a waterborne and foodborne pathogen that can damage human health. The exopolysaccharides (S-EPS) produced by *S. flexneri* CMCC51574 were found to promote biofilm formation and virulence. In this research, the crude S-EPS produced by *S. flexneri* CMCC51574 were separated into three main different fractions, S-EPS 1-1, S-EPS 2-1, and S-EPS 3-1. The structure of the *S*-ESP 2-1 was identified by FT-IR, ion chromatography analysis, methylation analysis, and NMR analysis. The main chain of S-EPS 2-1 was α-Manp-(1 → 3)-α-Manp-[(1 → 2,6)-α-Manp]_15_-[(1 → 2)-Manf-(1→]_8_; there were two branched-chain R1 and R2 with a ratio of 4:1, R1: α-Manp-(1 → 6)- and R2: α-Manp-(1 → 6)- Glc-(1 → 6)- were linked with (1 → 2,6)-α-Manp. It was found that S-EPS 2-1 exhibited the highest promoting effect on biofilm formation of *S. flexneri*. The S-EPS 2-1 was identified to interact with extracellular DNA (eDNA) of *S. flexneri*, indicating that the S-EPS 2-1 was the specific polysaccharide in the spatial structure of biofilm formation. Our research found the important role of S-EPS in *S. flexneri* biofilm formation, which will help us to understand the underlining mechanisms of the biofilm formation and find effective ways to prevent *S. flexneri* biofilm infection.

## Introduction

Bacteria exhibit two modes of growth: the free-living planktonic mode or the sessile, surface-attached mode within biofilms, which are structured communities encased in a self-produced polymer matrix mainly including exopolysaccharide, protein, and extracellular DNA (eDNA) (Rabin et al., 2015; Kim and Lee, 2016; Zhao et al., 2017; Rumbaugh and Sauer, 2020). The extracellular polymer matrix can provide the crucial architectural support and protect the microbial communities from the adverse external environment like antibiotics, over-acidic conditions, and so on (Flemming and Wingender, 2010; Jung et al., 2013; Algburi et al., 2017; Karygianni et al., 2020). Once pathogens form the biofilm, it will cause long-term continuous infection and be difficult to exterminate, which is common in daily living, food processing, and medical environments.

Antibiotics are commonly the first treatment for bacterial diseases. However, the pathogen can further enhance its tolerance to antibiotics by forming biofilm (Jiang et al., 2011; Hall and Mah, 2017; Roy et al., 2018). It is possible for pathogens to gradually become drug-resistant bacteria. Many epidemics caused by pathogens become difficult to control and have a high rate of recurrence due to the presence of pathogens biofilm (Venkatesan et al., 2015). Therefore, it is extremely important to explore the methods to exterminate the pathogen’s biofilm, and to explore the potential mechanism of pathogen biofilm formation.

*Shigella flexneri* is a Gram-negative, rod-shaped, facultative anaerobic pathogen that is a pathovar of *Escherichia coli* and is prevalent in developing countries (Anderson et al., 2016; Nisa et al., 2020). *S. flexneri* can invade human intestinal epithelial cells and spread from cell to cell, cause inflammation and damage to the mucosa of the intestinal epithelial cells, and lead to diarrhea, blood in the stool, and other symptoms in infected people (Agaisse, 2016). *S. flexneri* is recognized as waterborne and foodborne pathogens, seriously affecting food hygiene and human health (Fakruddin et al., 2017; Jneid et al., 2020; Fan et al., 2021). Foods are the widespread sources of *S. flexneri* contamination, including fruits, vegetables, a wide variety of meats, and fresh milk. Moreover, drug-resistant strains were commonly isolated from contaminated food or clinical environments (Ahmed and Shimamoto, 2015; Shahin et al., 2019). This may be ascribed to the high antibiotic resistance of *S. flexneri* biofilm. It was reported that the bile salts could promote the *S. flexneri* to form the strengthened biofilm and stimulate the expression of the virulence factors (Nickerson et al., 2017; Köseoğlu et al., 2019); at the same time, AcrAB efflux pump of *S. flexneri* can also help *S. flexneri* resist bile salt damage. Therefore, *S. flexneri* can cause human dysentery at low-level biomass. To prevent continued cross-infection due to the biofilm formation of *S. flexneri*, we need to explore the underlying mechanism of the *S. flexneri* biofilm formation and analyze the main components of *S. flexneri* biofilm.

Our previous studies Song et al. (2020) showed that the S-EPS (*S. flexneri* produced exopolysaccharide) could significantly promote the biofilm formation of *S. flexneri*. The EPS produced by bacteria are usually mixtures of different polysaccharides (Sun and Zhang, 2021). Therefore, we want to identify the specific EPS fraction that promotes the *S. flexneri* biofilm formation. In this research, the S-EPS was purified by the DEAE–Sepharose Fast Flow and Sepharose CL-6B to obtain the different single EPS components. The biofilm formation of *S. flexneri* treated by different single EPS components was assessed. Then, the structure analysis of the main active component was performed by ion chromatography, methylation analysis, and NMR spectrum. Finally, the key role of the main active single component in the structure of the *S. flexneri* biofilm was investigated. This research aimed to explore the effect of polysaccharides on *S. flexneri* biofilm formation and provide fundamental data for controlling *S. flexneri* biofilm formation.

## Materials and Methods

### Strains and Growth Medium

The *S. flexneri* CMCC51574 was stored in Luria-Bertani (LB) medium (Land Bridge, China) containing 25% glycerol at −80°C in Dalian probiotics function research key laboratory and cultured in LB medium at 37°C for 24 h.

### Extraction of *S. flexneri*-Exopolysaccharides

The extraction of *S. flexneri* EPS (S-EPS) was performed by the following method. The 5 L of fresh LB medium was inoculated with 2% overnight cultured *S. flexneri* LB suspension and incubated at 37°C for 24 h. After incubation, *S. flexneri* bacteria cells were removed by centrifugation (10,000 × *g* for 10 min at 4°C, CR21N, HITACHI, Ibaraki, Japan) to obtain a bacterial cell-free supernatant. The supernatant was concentrated by rotary evaporation to 1/5 of the original volume, and then the trichloroacetic acid was added to the concentrates to a final concentration of 4% (w/v), and the mixture was stirred for 30 min at room temperature. Precipitated proteins in the mixture were removed by centrifugation (10,000 × *g*, 4°C, 10 min). Then, two volumes of chilled absolute ethyl alcohol were added to the supernatant and stored at 4°C for 24 h to precipitate EPS. After centrifugation (10,000 × *g* for 10 min at 4°C), the obtained EPS precipitates were collected and dissolved in deionized water and dialyzed (MW cutoff 8,000–14,000 Da, Solarbio) against ultrapure water for 24 h at 4°C, and the ultrapure water was changed every 8 h. The dialyzed retentate was lyophilized to obtain the crude S-EPS. The crude S-EPS used in subsequent purification experiments were obtained from five times repeat extraction.

### Purification of *S. flexneri*-Exopolysaccharides

The purification of crude S-EPS was performed by the AKTA protein purification system (GE Healthcare, Chicago, IL, United States). The crude S-EPS solution (20 mg/ml and 5 ml) was separated by a chromatographic column (D 2.6 cm × 28 cm) filled with DEAE–Sepharose Fast Flow (GE Healthcare, Chicago, IL, United States). The samples were eluted with deionized water from 0 to 240 ml and 0.1 M NaCl with the linear gradient variation from 240 to 480 ml at a flow rate of 2 ml/min. Every 5 ml of elution was collected automatically, and the carbohydrate content was determined by the phenol–sulfuric acid method described in Section “Phenol–Sulfuric Acid Method.” The obtained fractions were lyophilized and further purified by the Sepharose CL-6B gel column (D 1.6 cm × 100 cm) and eluted with deionized water at a flow rate of 0.5 ml/min. A fraction containing purified S-EPS was collected, dialyzed, and lyophilized. The molecular weight of the purified S-EPS sample was calculated by using a standard curve. Gel Filtration Calibration Kit HMW (43, 75, 158, 440, and 669 kDa) (GE Healthcare, Chicago, IL, United States) was used as the Mw standard, and the elution volume was linear to the Lg Mw of standard.

### Structure Analysis of the Main Component of *S. flexneri*-Exopolysaccharides

#### The Functional Group Analysis by Fourier-Transform Infrared Spectroscopy

Fourier-transform infrared (FT-IR) spectra were recorded from the sample in the KBr pellet on the FT-IR spectrophotometer (PerkinElmer, Waltham, MA, United States). All the FT-IR analyses of EPS fractions were performed in the region of 4,000–500 cm^–1^.

#### Purity Detection of Polysaccharide

S-EPS samples were analyzed by the Waters e2695 HPLC system with Waters 2424 Evaporative Light Scattering Detectors (ELSD). The analytical column was TSK gel G4000PW_XL_ column (7.8 mm × 30 cm, 10 μm). The injection volume was 20 μl. Elution was carried out at a flow rate of 0.8 ml/min at 25°C. The mobile phase was ultrapure water.

#### Monosaccharide Composition Analysis

The monosaccharide composition was determined by ion chromatography analysis. The monosaccharide standards (fucose, rhamnose, arabinose, galactose, glucose, xylose, mannose, fructose, ribose, galacturonic acid and glucuronic acid, galactosamine hydrochloride, glucosamine hydrochloride, *N*-Acetyl-D glucosamine, L-guluronic acid, and D-mannuronic acid) were dissolved into ultrapure water as the mixed standard, and the final concentration was 10 mg/ml.

The EPS sample was added to an ampoule bottle and hydrolyzed by 3 M trifluoroacetic acid (TFA) for 3 h at 120°C, and the acid hydrolysis solution was accurately absorbed and transferred to the tube for nitrogen blow drying. Then, EPS hydrolyzed was re-dissolved in the ultrapure water and centrifuged (10,000 × *g*, 5 min) to obtain the supernatant. The supernatant was analyzed by ICS5000 (Thermo Fisher, Waltham, MA, United States). IC column: Dionex CarboPac PA 20 (3 mm × 150 mm); Mobile phase: A: H_2_O; B: 15 mM NaOH; C: 15 mM NaOH and 100 mM NaOAc; Flow rate: 0.3 ml/min; Injection volume: 5 μl; Column temperature: 30°C; Detector: electrochemical detector. The elution conditions are shown in Supplementary Table 1.

#### Methylation Analysis

The polysaccharide sample was methylated by a Methylation kit (Borui Saccharide, Jiangsu, China). Then, methylated polysaccharides were added into 1 ml of 2 M trifluoroacetic acid (TFA) for hydrolysis at 120°C for 90 min, evaporated with a rotary evaporator. The residue was re-dissolved with 2 ml of ultrapure water, and 60 mg of sodium borohydride was added into the solution for an 8-h reaction. The solution was neutralized with glacial acetic acid, concentrated by rotary evaporation, and dried at 101°C in the oven. Then, 1 ml of acetic anhydride was added for acetylation at 100°C for 1 h and cooled. Three milliliters of toluene was added, and the solution was dried with a rotary evaporator; this process was repeated five times to remove excess acetic anhydride. The acetylated product was dissolved in 3 ml of CH_2_Cl_2_ and transferred to a separation funnel. The ultrapure water was added; after sufficient shaking, the upper aqueous phase was removed. The process was repeated four times. The excess moisture of CH_2_Cl_2_ layer was dried with anhydrous sodium sulfate, and volume of supernatant was diluted to 10 mL by CH_2_Cl_2_, the sample was prepared for analysis. The samples were determined by gas chromatography-mass spectrometry with Shimadzu GCMS-QP 2010 (Japan).

GC-MS conditions: RXI-5 SIL MS column (30 mm × 0.25 mm × 0.25 μm). The programmed temperature rise condition was as follows: the initial temperature was 120°C, and the temperature was raised 3°C/min until 250°C and kept for 5 min. The inlet temperature was 250°C, the detector temperature was 250°C, the carrier gas was helium, and the flow rate was 1 ml/min.

#### NMR Analysis

The NMR spectrum was recorded by a Bruker Avance III 400 MHz NMR (Bruker Group, Switzerland). The 10-mg EPS sample was dissolved in 500 μl of D_2_O. The NMR spectrum of ^1^H, ^13^C, Dept 135, ^1^H-^1^H correlated spectroscopy (COSY), ^1^H-^13^C heteronuclear single-quantum coherence (HSQC), and heteronuclear multiple bond coherence (HMBC) was determined at room temperature.

### The Effect of Different Fractions of *S. flexneri*-Exopolysaccharides on the Biofilm Formation of *S. flexneri*

The effect of different fractions of S-EPS on *S. flexneri* biofilm formation was studied by the crystal violet staining method. The *S. flexneri* was cultured in LB medium and incubated at 37°C for 24 h. The concentration of *S. flexneri* was adjusted by fresh LB medium to 2 × 10^6^ CFU/ml. Then, 95 μl of the bacteria suspension and 5 μl of each fraction of S-EPS solution [S-EPS 1-1, 2-1, and 3-1 were dissolved in LB medium to 1.0, 2.0, and 4.0 mg/ml, respectively, and filtered with 0.22-μm sterile filtration membrane (Millex GP, United States)] were added into an optically clear flat-bottom 96-well plate (Costar 3599, Corning, Corning, NY, United States), and the control group just contained 95 μl of the bacteria suspension and 5 μl of fresh LB. The plates were covered and incubated aerobically at 37°C for 24 h. After incubation, the content of each well was removed, and each well was washed thrice with 200 μl of sterile PBS (pH 7.3) (Land Bridge, China) to remove non-adherent bacteria. The remaining attached bacteria were fixed with 200 μl of 95% methanol for 15 min, and the plates were emptied and left to dry. Then, each well was stained with 200 μl of 2% crystal violet for 5 min. The excess dye was removed by washing the plate with deionized water. After the plates were air-dried, the dye bound to the adherent cells was re-solubilized with 160 μl of 33% (v/v) glacial acetic acid. Aliquots of the solubilized dye from each well (125 μl) were transferred to a separate well in an optically clear flat-bottom 96-well plate, and the optical density (OD) was measured at 590 nm by Multiskan GO 1510 (Thermo Fisher, Vantaa, Finland). Results for this test were given as biofilm formation ratio (BF ratio) applying the following formula: BF ratio = OD_assay_/OD_control_. The biofilm formation ratio for each independent experiment was calculated by averaging the six replicate wells in each group. The data point was averaged from three independent repeat experiments and the standard deviation (SD) was calculated.

### The Effect of Purified *S. flexneri*-Exopolysaccharides on the Growth of *S. flexneri*

The growth curve of *S. flexneri* cultured in LB medium with purified S-EPS was determined by using the Automated Microbiology Growth Curve Analysis System FP-1100-C (Thermo, Vantaa, Finland). Briefly, 190 μl of the bacteria suspension in LB medium (2 × 10^6^ CFU/ml) and 10 μl of the purified S-EPS solution [S-EPS 1-1, 2-1, and 3-1 were dissolved in LB medium to 1.0, 2.0, and 4.0 mg/ml, respectively, and filtered with a 0.22-μm sterile filtration membrane (Millex GP, United States)] were added into Honeycomb 2 Sterilized (Oy Growth Curves Ab Ltd., Helsinki, Finland), and the control group just contained 190 μl of the bacteria suspension and 10 μl of the fresh LB. The OD_600_ was determined every 2 h at 37°C for 24 h. Each data point was averaged from five replicate wells and the standard deviation (SD) was calculated.

### Biofilm Observation by Scanning Electron Microscope and Fluorescence Microscopy

The biofilm observation by scanning electron microscope (SEM) was performed as follows (Gowrishankar et al., 2016). The 1.8 ml of 2 × 10^6^ CFU/ml bacteria suspension and 0.2 ml of 4 mg/ml purified S-EPS solution were added into an optically clear flat-bottom 6-well plate (Costar 3506, Corning, Corning, NY, United States). The biofilm was allowed to form on cover glass pieces (1 cm × 1 cm) placed in wells of the plate at 37°C for 24 h. After biofilm formed, the glass pieces were washed with PBS (pH 7.3) to remove all non-adherent bacteria. The formed biofilm on the glass pieces was fixed with 2.5% glutaraldehyde in PBS (pH 7.3) for 2 h. The glass pieces were washed in 0.1 M sodium acetate buffer (pH 7.3). Samples were subsequently washed in distilled water and dehydrated at increasing concentrations of ethanol (20, 50, 70, 90, and 100%) for 5 min each. After ethanol dehydration, the glass pieces were air-dried at room temperature. The biofilm was observed by electron scanning electron microscopy at 5,000 × magnification after gold spraying.

The observation by fluorescence microscope (FM) was performed as follows (Kang et al., 2018). The 1.8 ml of 2 × 10^6^ CFU/ml bacteria suspension and 0.2 ml of 4 mg/ml purified S-EPS solution were added into an optically clear flat-bottom 6-well plate (Costar 3506, Corning, Corning, NY, United States). The plate was cultured at 37°C for 24 h to form the biofilm. After that, the wells were gently washed thrice with PBS (pH 7.3) to remove non-adherent bacteria. Then, the calcofluor white stain was added to each well with equal volumes of 10% potassium hydroxide for 1 min [the Gelgreen Nucleic Acid Gel Stain (Biotium, Fremont, CA, United States) was diluted by ultrapure water and directly used to stain the sample for 1 min]. The dye solution was discarded and the stained biofilm was observed using an inverted fluorescence microscope (Olympus IX73, Japan).

### The Analysis of the Role of Polysaccharide and eDNA in the Biofilm Formation of *S. flexneri*

#### Identification of Monosaccharide Composition of Polysaccharides in Extracellular Polymer Matrix

Extraction of the extracellular polymer matrix of *S. flexneri* was carried out by heating method (Dai et al., 2016). Briefly, the 5 L of fresh LB medium was inoculated with 2% overnight cultured *S. flexneri* LB suspension and incubated at 37°C for 24 h. After incubation, *S. flexneri* bacteria cells were collected by centrifugation (10,000 × *g* for 10 min at 4°C, CR21N, HITACHI, Japan). The *S. flexneri* cells were washed once with PBS (pH 7.3) and re-suspended in 250 ml of 0.9% NaCl solution, and the suspension was incubated at 60°C for 30 min in a water bath. The supernatant was collected by centrifugation (10,000 × *g* for 10 min at 4°C, 5804R, Eppendorf, Germany). The polysaccharide in the supernatant was extracted by the method described in Section “Extraction of *S. flexneri*-Exopolysaccharides” and named SS-EPS (*S. flexneri* cell surface polysaccharide).

The monosaccharide composition analysis of SS-EPS was performed by the HPLC method (Song et al., 2020).

#### The Effect of DNase I on the Formed Biofilm of *S. flexneri*

The concentration of *S. flexneri* was adjusted by fresh LB medium to 2 × 10^6^ CFU/ml. Then, the *S. flexneri* suspension was added into an optically clear flat-bottom 96-well plate (Costar 3599, Corning, Corning, NY, United States). The plates were covered and incubated aerobically at 37°C for 24 h. After incubation, the content of each well was removed, and each well was washed thrice with 200 μl of sterile PBS (pH 7.3) (Land Bridge, China) to remove non-adherent bacteria. Then, the different concentrations (0.5, 1.0, 2.0, and 4.0 mg/ml) of DNase I (1,500 units/mg) (Solarbio, China) solution (dissolved in sterile deionized water) were added to the wells, respectively, and sterile deionized water was added into the wells of the control group. The plate was incubated aerobically at 37°C for 1 h. After that, the biofilm formation ratio was determined as described in Section “The Effect of Different Fractions of *S. flexneri*-Exopolysaccharides on the Biofilm Formation of *S. flexneri*.” The data point was averaged from three independent repeat experiments, and the standard deviation (SD) was calculated. Then, DNase I-treated biofilm formation was prepared in an optically clear flat-bottom 6-well plate (Costar 3506, Corning, Corning, NY, United States), and Gelgreen Nucleic Acid Gel Stain was used to observe the *S. flexneri* as described in Section “Biofilm Observation by Scanning Electron Microscope and Fluorescence Microscopy.”

#### The Changes of pH in the Medium and Polysaccharide Production of the *S. flexneri* Surface

The 4.75 ml of 2 × 10^6^ CFU/ml bacteria suspension and 0.25 ml of 4 mg/ml S-EPS 2-1 solution (ratio, 19:1, v/v) were co-cultured in the tubes at 37°C for 2, 4, 6…, 20, 22, 24 h; the pH of the medium in each group was detected.

Then, the *S. flexneri* in the 12-, 16-, 20-, and 24-h groups were collected by centrifugation (10,000 × *g*, 10 min, 4°C). The *S. flexneri* cells were washed once with PBS (pH 7.3) and adjusted the concentration to OD_600_ 2.0 by using 0.9% NaCl solution, and then 1.0 ml of the suspension was collected and incubated at 60°C for 30 min in a water bath. The supernatant was collected after centrifugation (10,000 × *g*, 10 min, 4°C). The polysaccharide content in the supernatant of *S. flexneri* was determined by the phenol–sulfuric acid method described in Section “Phenol–Sulfuric Acid Method.” The experiments were repeated three times independently.

For biofilm observation at different time points by the fluorescence microscope, the 1.8 ml of 2 × 10^6^ CFU/ml bacteria suspension and 0.2 ml of 4 mg/ml S-EPS 2-1 solution were added into an optically clear flat-bottom 6-well plate (Costar 3506, Corning, Corning, NY, United States). The plate was incubated at 37°C for 12, 16, 20, and 24 h, and then biofilm formation was observed by FM as described in Section “Biofilm Observation by Scanning Electron Microscope and Fluorescence Microscopy”; Gelgreen Nucleic Acid Gel Stain was used to observe the *S. flexneri* biofilm.

#### The Interaction Between *S. flexneri*-Exopolysaccharides 2-1 and DNA

The interaction between S-EPS 2-1 and DNA was determined by nucleic acid electrophoresis experiment, fluorescence spectra, and isothermal titration calorimetry (ITC). In brief, the DNA of *S. flexneri* was extracted by the TIANamp Bacteria DNA Kit (Tiangen, China). The S-EPS 2-1 sample was dissolved in the double-distilled H_2_O (dd H_2_O) at 4 mg/ml. The S-EPS 2-1 solution was co-incubated with equivalent 10 μg/ml of DNA solution (50 μl) at 37°C for 30 min with different pH conditions (pH 7.0 and 6.0); the pH was adjusted by 0.1 mol HCl. After that, the sample was analyzed by 1% agarose gel electrophoresis with Gelgreen Nucleic Acid Gel Stain (Biotium, Fremont, CA, United States). The fluorescence spectra were determined by the Shimadzu RF-6000 (Japan). The EPS and DNA sample was prepared and co-incubated as described above, and the Gelgreen Nucleic Acid Gel Stain was added before analysis. The fluorescence scanning was performed from 250 to 350 nm, and the excitation wavelength was 300 nm.

Isothermal titration calorimetry was performed as follows: 4 μg/ml of S-EPS 2-1 sample and 16 μg/ml of *S. flexneri* DNA were prepared in double-distilled H_2_O (dd H_2_O) and the pH was adjusted to 6.0 by 0.1 M HCl, and each solution was degassed. ITC measurements were performed with Affinity ITC (TA, United States). Titrations were carried out with 150 μl in the syringe and 350 μl in the cuvette. Each titration experiment consisted of 4-μl injections with 180-s intervals between each injection at 25°C, and the titration experiment was performed for a total of forty times injections. The ITC data were analyzed using Nano Analyze and fitted using the independent model.

### Phenol–Sulfuric Acid Method

Briefly, 25 μl of 6% phenol and 50 μl of samples were added and mixed in the optically clear flat-bottom 96-well plate (Costar 3599, Corning, Corning, NY, United States), and then 125 μl of concentrated sulfuric acid was added directly into the solution. The mixture was blown with a pipette, and allowed to stand for 30 min at room temperature. The optical density (OD) was measured at 490 nm by Multiskan GO 1510 (Thermo Fisher, Vantaa, Finland). The OD_490_ indicated the yield of polysaccharides.

### Statistical Analysis

SPSS 20.0 software was used for statistical analysis, and the difference significance test was conducted by single-factor variance analysis (ANOVA), least-significant difference (LSD), and Duncan multiple range test. All data are expressed as mean ± standard error; *p* < 0.05 indicates significant difference.

## Results

### Purification of *S. flexneri*-Exopolysaccharides

**FIGURE 1:**
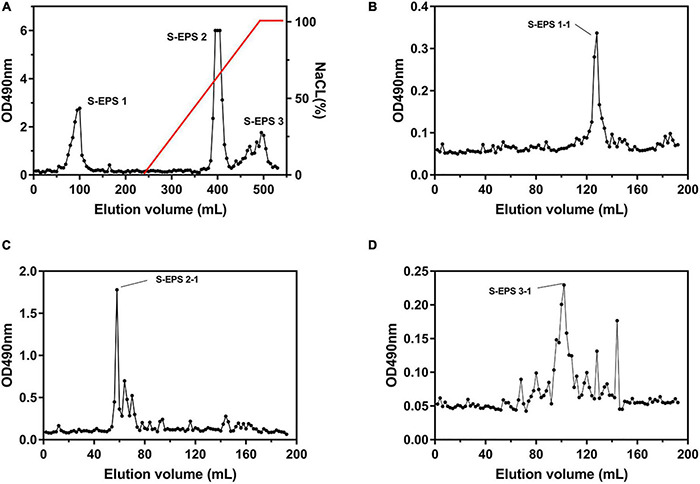
Elution curve of **(A)** S-EPS eluted by DEAE Sepharose Fast Flow, **(B)** S-EPS 1-1, **(C)** S-EPS 2-1, and **(D)** S-EPS 3-1 eluted by Sepharose CL-6B.

**FIGURE 2:**
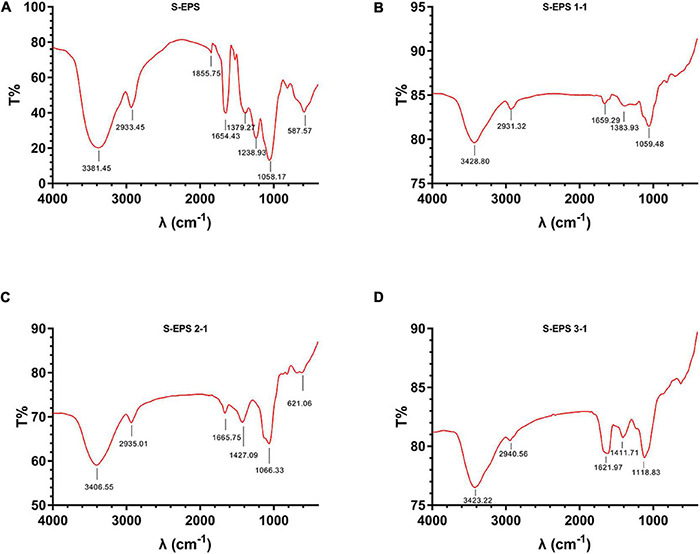
FT-IR spectra of **(A)** S-EPS, **(B)** S-EPS 1-1, **(C)** S-EPS 2-1, and **(D)** S-EPS 3-1.

The crude S-EPS was separated into S-EPS 1, S-EPS 2, and S-EPS 3 by the ion-exchange chromatography. As shown in Figure 1A, S-EPS 1 was eluted directly by the deionized water, indicating that S-EPS 1 was a polysaccharide without a negative charge. In contrast, S-EPS 2 and S-EPS 3 were eluted by NaCl solution, indicating that S-EPS 2 and S-EPS 3 were electronegative, and might possess the uronic acid. Subsequently, three kinds of S-EPS (S-EPS 1-1, S-EPS 2-1, and S-EPS 3-1) with uniform Mw (molecular weight) were obtained after being separated by size exclusion chromatography, as shown in Figures 1B–D. The Mw of S-EPS 1-1, S-EPS 2-1, and S-EPS 3-1 was determined as 82, 585, and 159 kDa (Supplementary Figure 1 and Supplementary Table 2). The FT-IR spectra of the EPS component are shown in Figure 2, the peak at 3,400 cm^–1^ was the stretching vibration absorption of -OH, and the peak at 2,930 cm^–1^ was the absorption peak of C-H. The peak at 1,400 cm^–1^ was the variable angle vibration of C-H. C = O had an absorption peak at 1,590 cm^–1^. The peak at 1,200–1,000 cm^–1^ was caused by two kinds of C-O stretching vibration (Thambiraj et al., 2018; Xu et al., 2018). It was further confirmed that the EPS component had the characteristic FT-IR spectra of polysaccharides.

### Structure Analysis of the Main Component of *S. flexneri*-Exopolysaccharides

The S-EPS 2-1 was regarded as the highest content of polysaccharide in the crude S-EPS and the structure of S-EPS 2-1 was determined.

#### Monosaccharide Composition Analysis

**FIGURE 3:**
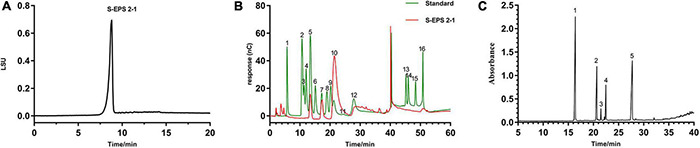
**(A)** The HPSEC analysis of S-EPS 2-1. **(B)** The ion chromatography analysis of S-EPS 2-1. (1) Fuc, (2) GalN, (3) Rha, (4) Ara, (5) GlcN, (6) Gal, (7) Glc, (8) GlcNAc, (9) Xyl, (10) Man, (11) Fru, (12) Rib, (13) GalA, (14) GulA, (15) GlcA, (16) ManA. **(C)** Methylation analysis of S-EPS 2-1 by GS-MS. (1) 2,3,4,6-Me4-Manp; (2) 3,4,6-Me3-Manf; (3) 2,4,6-Me3-Manp; (4) 2,3,4-Me3-Glcp; (5) 3,4-Me2-Manp.

As shown in Figure 3A, the S-EPS 2-1 was determined as a single peak by HPSEC, indicating that S-EPS 2-1 was a homogeneous polysaccharide component. The monosaccharide composition analysis was determined by ion chromatography, as shown in Figure 3B and Table 1. The S-EPS 2-1 was composed of mannose, glucose, and glucosamine hydrochloride with molar percentage ratios of 0.88, 0.10, and 0.02, respectively.

**TABLE 1:** Monosaccharide compositions of S-EPS 2-1.

Name	RT	Molar ratio	Name	RT	Molar ratio
Fuc	5.717	0.000	Xyl	20.209	0.000
GalN	10.675	0.000	Man	21.434	0.880
Rha	11.359	0.000	Fru	24.367	0.000
Ara	12.042	0.000	Rib	27.884	0.000
GlcN	13.442	0.020	GalA	45.209	0.000
Gal	15.1	0.000	GulA	45.992	0.000
Glc	17.317	0.100	GlcA	48.409	0.000
GlcNAc	18.95	0.000	ManA	50.817	0.000

#### Methylation Analysis

The methylation analysis was performed to identify the glycosidic bond types. As shown in Figure 3C and Table 2, S-EPS 2-1 contained five kinds of glycosidic bonds, 1-linked mannose (pyran), 1,2-linked mannose (furan), 1,3-linked mannose (pyran), 1,6-linked glucose (pyran), and 1,2,6-linked mannose (pyran) with molar percentage ratios of 37.63, 18.86, 2.40, 8.82, and 32.29, respectively. The S-EPS 2-1 contained 91.18% of the glycosidic bond type of mannose, which was consistent with the monosaccharide analysis. The results indicated that the major backbone of S-EPS 2-1 might contain 1-linked mannose, 1,2-linked mannose, 1,3-linked mannose, 1,2,6-linked mannose, and the branched-chain containing 1,6-linked glucose and link with 1,2,6-linked mannose. The identification of specific glycosidic bond linkage was combined with NMR analysis.

**TABLE 2:** Type of linkage of S-EPS 2-1.

RT	Methylated sugar	Mass fragments (m/z)	Area (%)	Type of linkage
16.398	2,3,4,6-Me4-Manp	43, 71, 87, 101, 117, 129, 145, 161, 205	37.63	Manp-(1→
20.667	3,4,6-Me3-Manf	43, 71, 87, 99, 101, 113, 129, 145, 161, 189	18.86	→2)-Manf-(1→
21.474	2,4,6-Me3-Manp	43, 87, 101, 117, 129, 161, 201, 233	2.40	→3)-Manp-(1→
22.462	2,3,4-Me3-Glcp	43, 71, 87, 99, 101, 117, 129, 161, 173, 189, 233	8.82	→6-Glcp-(1→
27.731	3,4–Me2-Manp	43, 87, 99, 129, 189	32.29	→2,6)-Manp-(1→

#### NMR Analysis

**FIGURE 4:**
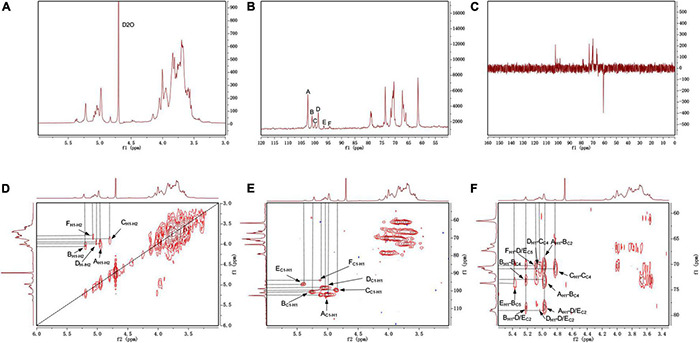
The NMR spectrum of S-EPS 2-1 in D_2_O. **(A)** ^1^H; **(B)** ^13^C; **(C)** DEPT-135; **(D)** ^1^H-^1^H COSY; **(E)** HSQC; **(F)** HMBC.

The NMR spectrum was recorded by a Bruker Avance III 400 MHz NMR. Anomeric proton and anomeric carbon signal ranges are 4.3–5.9 and 90–112, respectively. In the region, the numbers of signals usually correspond to the amount of anomeric proton and anomeric carbon, reflecting the numbers of sugar residues. Because there were many overlapped signals in the ^1^H spectrum (Figure 4A), ^13^C was used for the first step analysis of sugar residues (Figure 4B). We assumed that the signals in the anomeric carbon region were A, B, C, D, E, and F corresponding to signals at 102.59, 101.33, 99.84, 98.68, 96.62, and 94.47, representing each of the six kinds of possible glycosidic bond, respectively. The signals of anomeric proton directly linked to anomeric carbon corresponded one-to-one through HSQC (Figure 4E). The proton signals of ortho-position of the anomeric proton were located by the H-H COSY (Figure 4D). And the spectra of Dept 135 (Figure 4C) indicated that the carbon atoms with positive signal peak were primary or tertiary carbon atoms, and the carbon atoms with negative signal peak were secondary carbon atoms. Combined with references (Olennikov et al., 2010; Wang et al., 2014; Chatterjee et al., 2018; Malinowska et al., 2018) and methylation analysis, all of the proton-carbon-related signals are summarized in Table 3. The interrelationships between sugar residues were deduced by HMBC (Figure 4F), and the results are summarized in Table 4.

**TABLE 3:** Assignments of ^1^H and ^13^C NMR spectra for S-EPS 2-1.

	H1/C1	H2/C2	H3/C3	H4/C4	H5/C5	H6/C6
(A) α-Manp-(1→	4.98	4.00	3.83	3.54	3.41	3.81
	102.59	70.36	70.48	67.32	75.35	61.44
(B) →3)-α-Manp-(1→	5.22	4.05	3.66	3.70	3.38	3.69
	101.33	70.13	77.46	73.00	73.34	61.67
(C) →2)-Manf-(1→	4.83	3.84	3.59	3.94	3.77	3.58
	99.84	70.66	66.65	70.88	66.43	66.12
(D) →2,6)-α-Manp-(1→	5.04	3.95	3.78	3.56	3.54	3.64
	98.68	78.74	70.66	66.96	70.53	66.96
(E) →2,6)-α-Manp-(1→	5.39	3.95	3.78	3.56	3.54	3.64
	96.62	78.74	70.66	66.96	70.53	66.96
(F) →6)-α-Glcp-(1→	5.10	3.78	3.57	3.82	3.35	3.60
	94.47	70.57	61.17	65.90	70.45	66.96

**TABLE 4:** Glycosidic bonds related to each other.

H signal	C signal	Glycosidic link
H1 of →2,6)-α-Manp-(1→	C5 of →3)-α-Manp-(1→	→2,6-α-Manp-1→3-α-Manp-1→
H1 of →3)-α-Manp-(1→	C4 of →3)-α-Manp-(1→	→3-Manp-1→3-Manp-1→
H1 of →3)-α-Manp-(1→	C2 of →2,6)-α-Manp-(1→	→3-Manp-1→2,6-α-Manp-1→
H1 of →2,6)-α-Manp-(1→	C4 of →2)-Manf-(1→	→2,6-α-Manp-1→2-Manf-1→
H1 of →2,6)-α-Manp-(1→	C2 of →2,6)-α-Manp-(1→	→2, 6-α-Manp-1→2, 6-α-Manp-1→
H1 of α-Manp-(1→	C2 of →3)-α-Manp-(1→	α-Manp-1→3-α-Manp-1→
H1 of α-Manp-(1→	C2 of →2,6)-α-Manp-(1→	α-Manp-1→2, 6-α-Manp-1→
H1 of →2)-Manf-(1→	C2 of →2)-Manf-(1→	→2-Manf-1→2-Manf-1→

**FIGURE 5:**
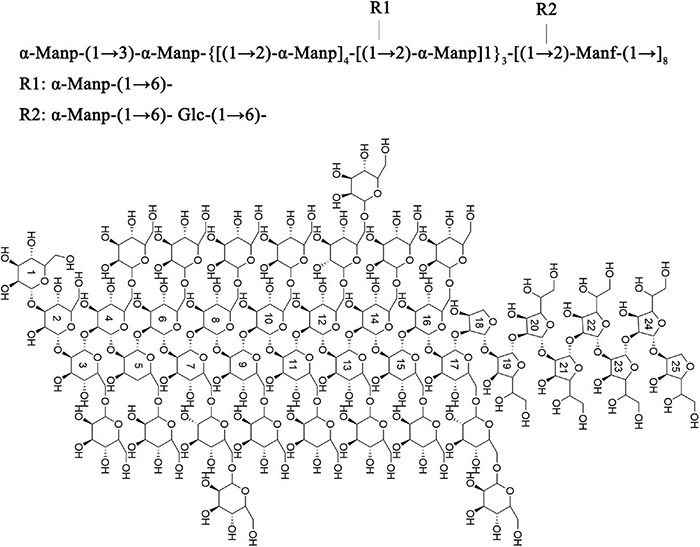
The structural formula of S-EPS 2-1.

Based on the above results, the main chain of S-EPS 2-1 was deduced as α-Manp-(1 → 3)-α-Manp-[(1 → 2,6)-α-Manp]_15_-[(1 → 2)-Manf-(1→]_8_, and there were two branched-chain R1 and R2 with a ratio of 4:1, R1: α-Manp-(1 → 6)- and R2: α-Manp-(1 → 6)- Glc-(1 → 6)-, as shown in Figure 5. The structural formula of S-EPS 2-1 was drawn with ChemDraw 20.0 software.

### The Effect of Purified *S. flexneri*-Exopolysaccharides on the Biofilm Formation of *S. flexneri*

**FIGURE 6:**
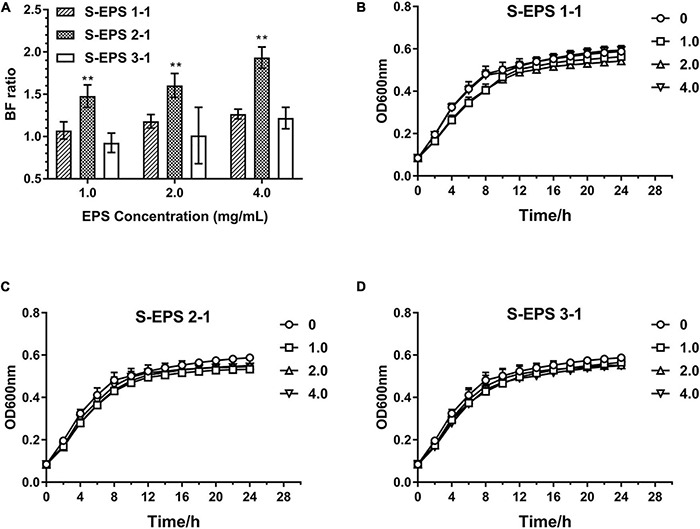
Biofilm formation ratio of *S. flexneri* after cultured with different concentrations of S-EPS 1-1, S-EPS 2-1, and S-EPS 3-1 (1.0, 2.0, and 4.0 mg/ml) for 24 h **(A)**; “**” indicated significant difference with other groups, *p* < 0.01. Growth curve of *S. flexneri* cultured with different concentrations of **(B)** S-EPS 1-1, **(C)** S-EPS 2-1, and **(D)** S-EPS 3-1 (1.0, 2.0, and 4.0 mg/ml) for 24 h.

**FIGURE 7:**
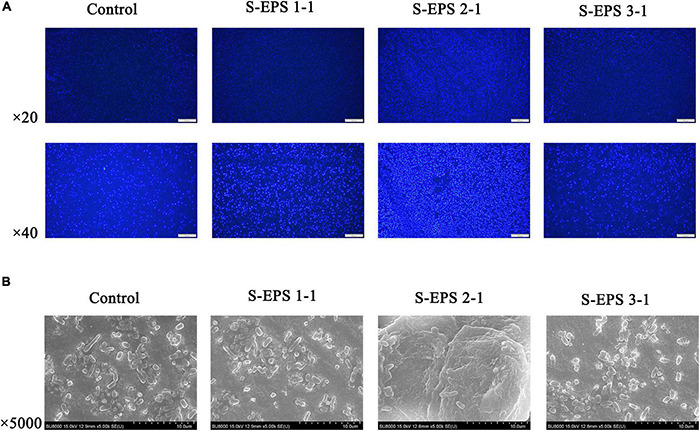
**(A)** Fluorescence microscopy image and **(B)** SEM images of *S. flexneri* biofilm formation treated with 4 mg/ml of different S-EPS fractions compared to control. Scale bars indicate 100 μm (20×) and 50 μm (40×) in **(A)**, and 10 μm in **(B)**.

As shown in Figure 6A, the S-EPS 1-1, S-EPS 2-1, and S-EPS 3-1 could promote the BF of *S. flexneri*. The BF ratio of the S-EPS 2-1 group was significantly more than that of the other two groups, *p* < 0.01. The growth curve of each group did not exhibit a significant difference, as shown in Figures 6B–D, indicating that the three kinds of S-EPS could not increase the number of *S. flexneri* during the culture process, which might provide *S. flexneri* more opportunities for adhesion or aggregation. The adhesion ability of *S. flexneri* on the tissue culture plate and cover glass was observed by the FM and SEM. As shown in Figure 7A, the fluorescence intensity and the density of the *S. flexneri* in the S-EPS 2-1 group were significantly higher than that of the other two groups, indicating that S-EPS 2-1 increased the adhesion numbers of *S. flexneri* on the polyethylene material. The SEM image showed that aggregation was formed in the S-EPS 2-1 group, while the *S. flexneri* adhesion number and colony number in the other two groups were not significantly different from the control group.

### S-EPS 2-1 and eDNA Are the Key Components in the Biofilm

**FIGURE 8:**
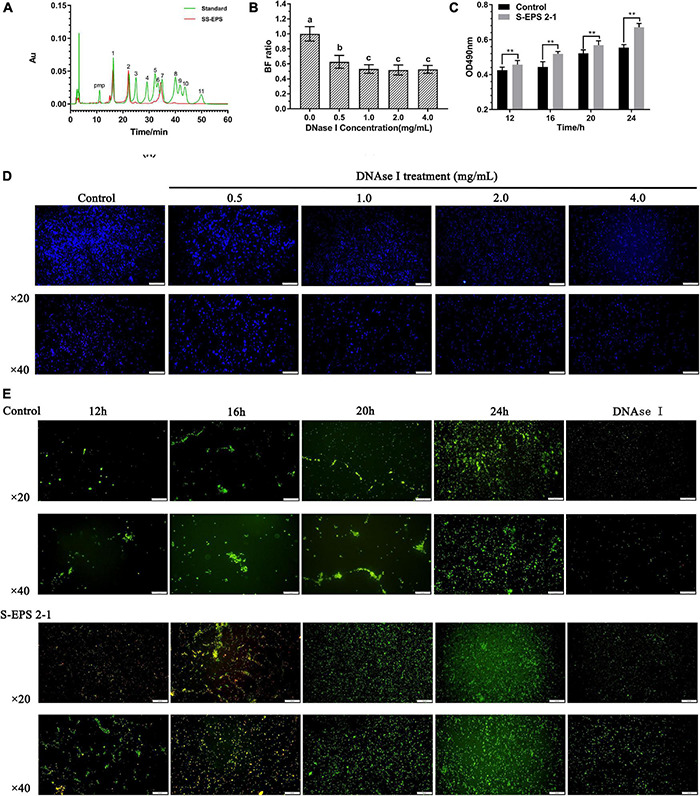
**(A)** HPLC chromatogram of SS-EPS and standards with peaks as follows: (1) Man, (2) Rha, (3) GlcA acid, (4) GalA, (5) GalN, (6) GlcN, (7) Glc, (8) Gal, (9) Xyl, (10) Ara, and (11) Fuc. **(B)** Biofilm formation ratio of *S. flexneri* after treated with different concentrations of DNase I for 1 h. **(C)** Polysaccharide content in the extracellular polymer matrix at different culture time points. **(D)** Fluorescence microscopy image of *S. flexneri* biofilm formation after treated with different concentrations of DNase I for 1 h; the biofilm was stained by calcofluor white stain. Scale bars indicate 100 μm (20×) and 50 μm (40×). **(E)** Fluorescence microscopy image of *S. flexneri* biofilm formation after treated with 4 mg/ml S-EPS 2-1 for 12, 16, 20, and 24 h; the biofilm was stained by Gelgreen stain. Scale bars indicate 100 μm (20×) and 50 μm (40×). “**” indicated the significant difference, *p* < 0.01. The different lowercase letters on the column indicated the significant difference, *p* < 0.05.

To confirm the existence of S-EPS 2-1 in the biofilm, we analyzed the monosaccharide composition of polysaccharide (SS-EPS) in the extracellular polymer matrix. As shown in Figure 8A, the SS-EPS was mainly composed of mannose, rhamnose, and glucose. However, S-EPS 2-1 was identified as mainly composed of mannose and glucose, and did not contain rhamnose. In the previous studies, the monosaccharide composition of crude S-EPS was identified as mainly composed of mannose and glucose, also not containing rhamnose (Song et al., 2020). We speculated that the rhamnose might be derived from lipopolysaccharide (LPS), because of the bacterial lysis leading to the dissolution of LPS in the extraction process of SS-EPS. Therefore, we could deduce that S-EPS 2-1 might be the major polysaccharide component in the extracellular polymer matrix of the *S. flexneri* biofilm. Furthermore, we found that the formed biofilm of *S. flexneri* could significantly be disrupted by the DNase I, as shown in Figure 8B. The phenomenon could be observed in the image of the fluorescence microscope (Figure 8D). There was a weaker fluorescence intensity in the DNase I-treated group than that of the control group, and the aggregations of *S. flexneri* were significantly reduced, indicating that the DNA was also the main component in the *S. flexneri* biofilm. Therefore, the S-EPS 2-1 and DNA played an important role in the biofilm formation of *S. flexneri*.

In our previous studies (Song et al., 2020), we confirmed that *S. flexneri* could form the biofilm after being cultured for 12 h, and the crude S-EPS could significantly promote biofilm formation of *S. flexneri* after being cultured for 20 h. In this research, we could observe that the adhesion number of *S. flexneri* in the S-EPS 2-1-treated group was more than that in the control group at different time points, and the polysaccharide content of the *S. flexneri* surface was significantly increased after being treated with S-EPS 2-1 (Figures 8C,E). Therefore, the S-EPS 2-1 might increase the aggregation ability and adhesion ability to the polystyrene surface.

### The Interaction Between Exopolysaccharides and DNA

**FIGURE 9:**
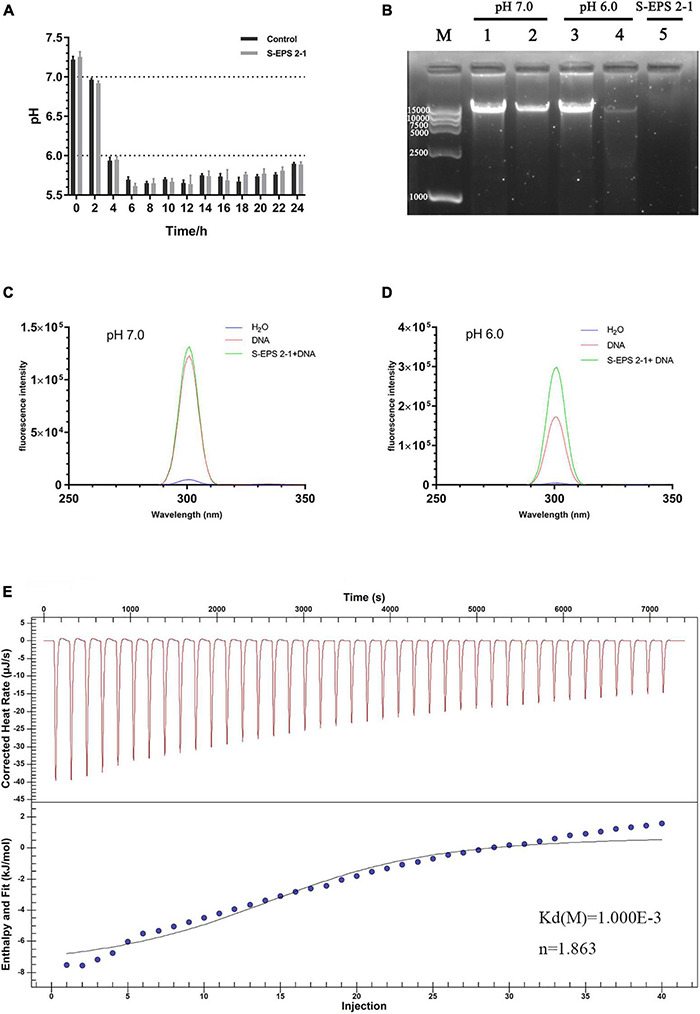
**(A)** The pH changes of the culture medium of *S. flexneri* during 24 h cultivation. **(B)** The nucleic acid electrophoresis analyzed the interaction between S-EPS 2-1 and DNA. Lane M, marker DL 15000; lanes 1 and 3: DNA only; lanes 2 and 4: EPS + DNA incubated at 37°C for 30 min; lane 5: S- EPS 2-1 only. The fluorescent spectra analyzed the interaction of EPS and DNA, **(C)** pH 7.0; **(D)** pH 6.0; **(E)** isothermal titration calorimetry measurement to determine the interaction between S-EPS 2-1 and DNA.

The DNA in the extracellular polymer matrix was usually derived from bacterial cell death and lysis in the interior of the microcolony, and DNA could bind with EPS to form the spatial structure of the biofilm depending on the pH (Jennings et al., 2015; Peng et al., 2019). Therefore, the interaction between S-EPS 2-1 and DNA of *S. flexneri* was analyzed at different pH conditions. The range of pH changes during *S. flexneri* cultured for 24 h was approximately from 7.20 to 5.80 (Figure 9A). Hence, we chose pH 7.0 and 6.0 conditions to determine the interaction between S-EPS 2-1 and DNA. As shown in Figure 9B, after the S-EPS 2-1 and DNA were co-incubated for 30 min at 37°C, the existence of a clear band (lane 2) could be observed, indicating that the DNA did not change or there might not be an interaction between S-EPS 2-1 and DNA at pH 7.0. However, under the condition of pH 6.0, it could be observed that the band nearly disappeared, and a weak band could still be seen (lane 4), indicating that there might be some kind of changes in the DNA or the interaction between S-EPS 2-1 and DNA. To figure out whether the degradation of DNA caused the bands to disappear, the fluorescence spectra of the solution of S-EPS 2-1 and DNA co-incubating at pH 6.0 were performed. The Gelgreen Nucleic Acid Gel Stain was used to bond with double-stranded DNA and fluoresce at an excitation wavelength of 300 nm. As shown in Figures 9C,D, after S-EPS 2-1 and DNA co-incubated at pH 6.0, the solution still showed a high fluorescence intensity, indicating that the DNA in the solution might not be degraded and still maintained its original double-stranded structure. The ITC experiment demonstrated that the S-EPS 2-1 could interact with DNA, the binding constant was 10^–3^, and the binding ratio was 1:1.863. Therefore, it was deduced that S-EPS 2-1 could interact with DNA to form the spatial structure of the *S. flexneri* biofilm.

## Discussion

In this research, we focused on the important role of specific polysaccharides and DNA in the spatial structure of biofilm formation of *S. flexneri*. The S-EPS was purified by the DEAE–Sepharose Fast Flow and Sepharose CL-6B to obtain three different polysaccharide fractions with uniform electronegative and molecular weight. We found that the fraction S-EPS 2-1 exhibited a higher promoting effect on the biofilm formation of *S. flexneri* than the other two polysaccharide components (Figure 6A). In the case that polysaccharides could not promote the growth of *S. flexneri* (Figures 6B–D), it was believed that polysaccharides might play a crucial role in the structure of the *S. flexneri* biofilm.

The polysaccharide was extensively reported to interact with the eDNA to form the skeleton of the bacteria biofilm. Hu et al. (2012) confirmed that eDNA of *Myxococcus xanthus* could bind to and colocalize with exopolysaccharides within the native extracellular matrices of starvation biofilms and fruiting bodies. The biofilm 3D structure of *Bacillus subtilis* showed that polysaccharides could interconnect with eDNA in the early stage of biofilm formation, and was mainly the structure material of the mature biofilm (Peng et al., 2019). Kanampalliwar and Singh (2020) reported that the interaction between eDNA and polysaccharides could contribute to the biofilm integrity of *Vibrio cholerae*. In this research, the S-EPS 2 could bond on the anion exchange column DEAE-Sepharose Fast Flow during the S-EPS purified process and S-EPS 2-1 was the main component of S-EPS 2, indicating that S-EPS 2-1 might have the negative charge (Figure 1). The cationic polysaccharide might be easier to interact with negatively charged eDNA by electrostatic interaction (Jennings et al., 2015). Our data suggested that the S-EPS 2-1 interacts with DNA at pH 6.0, but not at pH 7.0 (Figure 9). It might be ascribed to the positive charge of S-EPS 2-1 at pH 6.0, and the DNA could interact with S-EPS 2-1 by electrostatic interaction. The bacterial aggregation of *S. flexneri* after being treated with S-EPS 2-1 (Figure 7B) could be observed.

The monosaccharide composition analysis indicated that the S-EPS 2-1 was present in the extracellular polymer matrix of biofilm (Figures 3B, 8A). More adhesion and aggregation numbers of bacteria could be observed on the surface of polystyrene material in the S-EPS 2-1-treated group than those in the control group at different culture time points (Figure 8E). Our data suggested that DNase I could decrease the biofilm biomass, but not eliminate the biofilm. Therefore, S-EPS 2-1 might play an important role in the whole process of biofilm formation of *S. flexneri*. It was speculated that DNA and S-EPS 2-1 might provide adhesion ability for *S. flexneri* and jointly maintain the overall structure of the biofilm.

In addition to the important role in the structure of bacteria biofilm, the bacteria polysaccharides could also promote the biofilm formation as signaling molecules (Irie et al., 2012). *Pseudomonas aeruginosa* could secrete three kinds of polysaccharides, alginate, Pel, and Psl (Ryder et al., 2007). The alginate was widely present in the matrix of *P. aeruginosa* biofilm but was not necessary for biofilm formation, and mainly functioned as a virulence factor (Ryder et al., 2007; Wang et al., 2016). Pel and Psl are very important for the biofilm formation of *P. aeruginosa* (Ryder et al., 2007; Colvin et al., 2012; Jones and Wozniak, 2017). Pel was positively charged, and composed of 1 → 4 linked partially acetylated galactosamine and glucosamine, and was reported to interact with eDNA to maintain the structural stability of *P. aeruginosa* biofilm (Jennings et al., 2015; Jones and Wozniak, 2017). Psl is composed of D-mannose, D-glucose, and L-rhamnose, the main chain is (1 → 3)-β-D-Manp, and the branched chain is α-L-Rhap-(1 → 3)-β-D-Glcp-(1→) (Byrd et al., 2009; Jones and Wozniak, 2017). Psl could act as a signal to stimulate the production of intracellular secondary messenger molecule c-di-GMP that promotes biofilm formation, and the upregulated c-di-GMP could increase the extracellular polymer matrix secretion including Psl (Irie et al., 2012). Furthermore, Psl could also interact with eDNA to form the biofilm skeleton of *P. aeruginosa* (Wang et al., 2015). In this research, S-EPS could be purified into three kinds of single polysaccharide components, S-EPS 1-1, S-EPS 2-1, and S-EPS 3-1. Although the results indicated that S-EPS 2-1 had the highest positive effect on the BF of *S. flexneri*, S-EPS 1-1 and S-EPS 3-1 could also slightly promote the BF of *S. flexneri* (Figure 2). Therefore, S-EPS 1-1, S-EPS 2-1, and S-EPS 3-1 might function in BF as a signal-like molecule. Whether polysaccharides could regulate the expression of genes related to biofilm formation of *S. flexneri* also remained to be investigated. In future experimental plans, we will study the effect of polysaccharides on the gene expression level of *S. flexneri* by transcriptome.

The structure of polysaccharides of S-EPS 2-1 might be related to the formation of biofilm. According to the classification of homopolysaccharides and heteropolysaccharides (Sun and Zhang, 2021), S-EPS 2-1 was a heteropolysaccharide polymerized by abundant mannose and a little glucose. FimH gene encoded the minor fimbrial subunit, which was a D-mannose-specific adhesin (Liu et al., 2021). The FimH adhesin was important for the adhesion and infection abilities of many Gram-negative bacteria (Vanwetswinkel et al., 2014; Rafsanjany et al., 2015; Sauer et al., 2019). Uropathogenic *Escherichia coli* invasion was effectively mediated by the adhesion of FimH adhesin, which could recognize α-D-pyran mannose of high-mannose-type *N*-glycans on the surface of urothelial cells (Vanwetswinkel et al., 2014; Sauer et al., 2019). In this research, S-EPS 2-1 was found rich in mannose; therefore, we hypothesized that S-EPS 2-1 might also be recognized by FimH adhesin of *S. flexneri* to promote bacterial aggregation and adhesion.

Based on the characteristics of the *S. flexneri* biofilm, we can provide some suggestions for preventing biofilm formation. The structural integrity of biofilm can be damaged by the loss of polysaccharide and DNA interactions, but not eliminated thoroughly. It was extensively reported that saccharides, including oligosaccharides and polysaccharides, could inhibit the biofilm formation of pathogens by disrupting polysaccharide interactions with DNA or by acting as a spatial hindrance to interference interaction between the extracellular polymer matrix (Jiang et al., 2011; Craft and Townsend, 2018). Our previous study found that EPS (L-EPS) produced by *Lactobacillus plantarum* 12 could inhibit the biofilm formation of *S. flexneri*, and *S. flexneri* that lived in biofilm could be effectively killed using a combination of L-EPS with low-dose antibiotics (Song et al., 2020). Therefore, we recommend a combination of several reasonable methods to combat pollution caused by biofilm.

## Conclusion

The results showed that the S-EPS produced by *S. flexneri* were mixtures of fractions S-EPS 1-1, S-EPS 2-1, and S-EPS 3-1. The data demonstrated that S-EPS 2-1 could interact with DNA and significantly promote the biofilm formation of *S. flexneri*. S-EPS 2-1 was deduced as the specific polysaccharide in the spatial structure of *S. flexneri* biofilm formation. The structure of S-EPS 2-1 was identified, the main chain was α-Manp-(1 → 3)-α-Manp-[(1 → 2,6)-α-Manp]_15_-[(1 → 2)-Manf-(1→]_8_, and there were two branched-chain R1 and R2 with a ratio of 4:1, R1: α-Manp-(1 → 6)- and R2: α-Manp-(1 → 6)- Glc-(1 → 6)-. In future research, we will focus on the gene expression level of *S. flexneri* treated with different polysaccharides and explore the polysaccharide biosynthetic pathway of *S. flexneri*. Our research aims to reveal the important role of polysaccharides in *S. flexneri* biofilm formation and invasion ability and find effective ways to prevent *S. flexneri* biofilm infection and invasion.

## Data Availability Statement

The original contributions presented in the study are included in the article/Supplementary Material, further inquiries can be directed to the corresponding author/s.

## Author Contributions

YS, YT, and GM contributed to conception and designed of the study. YT was the host of the national nature Fund project. YS performed the experiments, statistical analysis, and wrote the first draft of the manuscript. MS and FM wrote sections of the manuscript. All authors contributed to manuscript revision, read, and approved the submitted version.

## Conflict of Interest

The authors declare that the research was conducted in the absence of any commercial or financial relationships that could be construed as a potential conflict of interest.

## Publisher’s Note

All claims expressed in this article are solely those of the authors and do not necessarily represent those of their affiliated organizations, or those of the publisher, the editors and the reviewers. Any product that may be evaluated in this article, or claim that may be made by its manufacturer, is not guaranteed or endorsed by the publisher.

## Supplementary Material

Supplementary Figure 1 Standard curve of molecular weight determination.
