# Supplementary figures and images for: The Chemical Structure Properties and Promoting Biofilm Activity of Exopolysaccharide Produced by Shigella flexneri
Source: Front Microbiol. 2022 Feb 4;12:807397. doi: 10.3389/fmicb.2021.807397 (PMC8854994; doi:10.3389/fmicb.2021.807397)

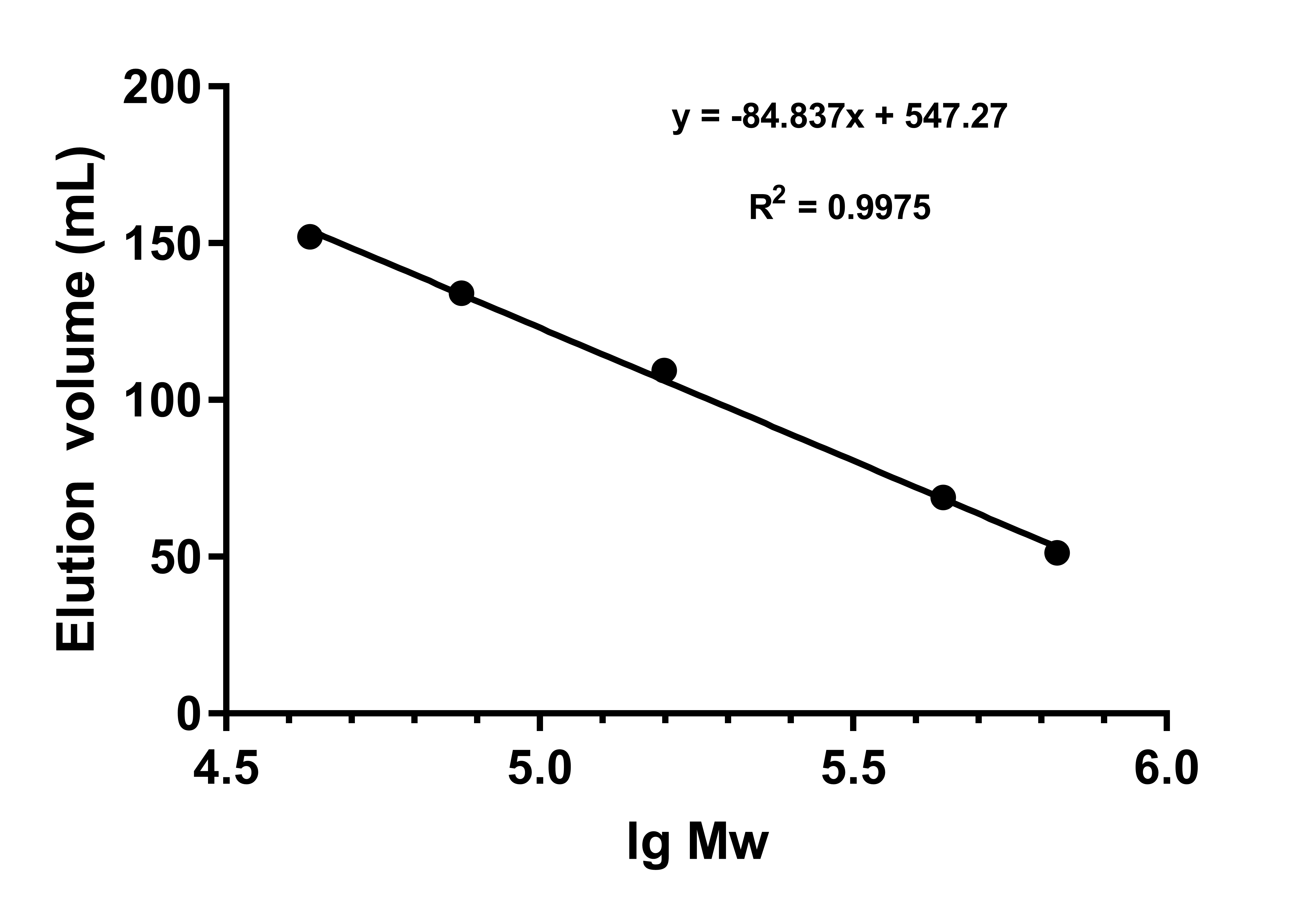

Supplement: Supplementary Figure 1 — Standard curve of molecular weight determination. [file Image_1.TIF]
