# Supplementary material for: The Chemical Structure Properties and Promoting Biofilm Activity of Exopolysaccharide Produced by Shigella flexneri
Source: Front Microbiol. 2022 Feb 4;12:807397. doi: 10.3389/fmicb.2021.807397 (PMC8854994; doi:10.3389/fmicb.2021.807397)
Supplement: Supplementary file 2 [file Data_Sheet_1.docx]

**Supplementary material**

**Table S1 Gradient elution conditions of ion chromatography analysis.**

| **Table Step** | Time (min) | A (%) | B(%) | C(%) |
| --- | --- | --- | --- | --- |
| 1 | 0.0 | 98.8 | 1.2 | 0 |
| 2 | 18.0 | 98.8 | 1.2 | 0 |
| 3 | 20.0 | 50 | 50 | 0 |
| 4 | 30.0 | 50 | 50 | 0 |
| 5 | 30.1 | 0 | 0 | 100 |
| 6 | 46.0 | 0 | 0 | 100 |
| 7 | 46.1 | 0 | 100 | 0 |
| 8 | 50.0 | 0 | 100 | 0 |
| 9 | 50.1 | 98.8 | 1.2 | 0 |
| 10 | 60.0 | 98.8 | 1.2 | 0 |

**Table S2 Molecular weight of different S-EPS fraction.**

| y = -84.837x + 547.27 R² = 0.9975 | S-EPS | | |
| --- | --- | --- | --- |
|  | S-EPS 1-1 | S-EPS 2-1 | S-EPS 3-1 |
| Ev (mL) | 130 | 58 | 106 |
| Mw (KDa) | 82 | 585 | 159 |
